# Supplementary material for: Aluminum Nitride Ultraviolet Light-Emitting Device Excited via Carbon Nanotube Field-Emission Electron Beam
Source: Nanomaterials (Basel). 2023 Mar 16;13(6):1067. doi: 10.3390/nano13061067 (PMC10053685; doi:10.3390/nano13061067)
Supplement: Supplementary file 1 [file nanomaterials-13-01067-s001.zip › nanomaterials-2264575-supplementary.pdf]

nanomaterials

MDPI

Supplementary Materials

Aluminum Nitride Ultraviolet Light–Emitting Device Excited via Carbon Nanotube Field–emission Electron Beam

Yangcheng Yu [1,†], Dong Han [2,†], Haiyuan Wei [1], Ziying Tang [1], Lei Luo [2], Tianzeng Hong [2], Yan Shen [2,*], Huying Zheng [1], Yaqi Wang [1], Runchen Wang [1], Hai Zhu [1,*] and Shaozhi Deng [2,*]

[1] State Key Laboratory of Optoelectronic Materials and Technologies, School of Physics, Sun Yat–Sen University, Guangzhou 510275, China
[2] State Key Laboratory of Optoelectronic Materials and Technologies, Guangdong Province Key Laboratory of Display Material and Technology, School of Electronics and Information Technology, Sun Yat–Sen University, Guangzhou 510275, China
* Correspondence: shenyan7@mail.sysu.edu.cn (Y.S.); zhuhai5@mail.sysu.edu.cn (H.Z.); stsdsz@mail.sysu.edu.cn (S.D.)
† These authors contributed equally to this work.

I. The Device Components and Operating Circuit

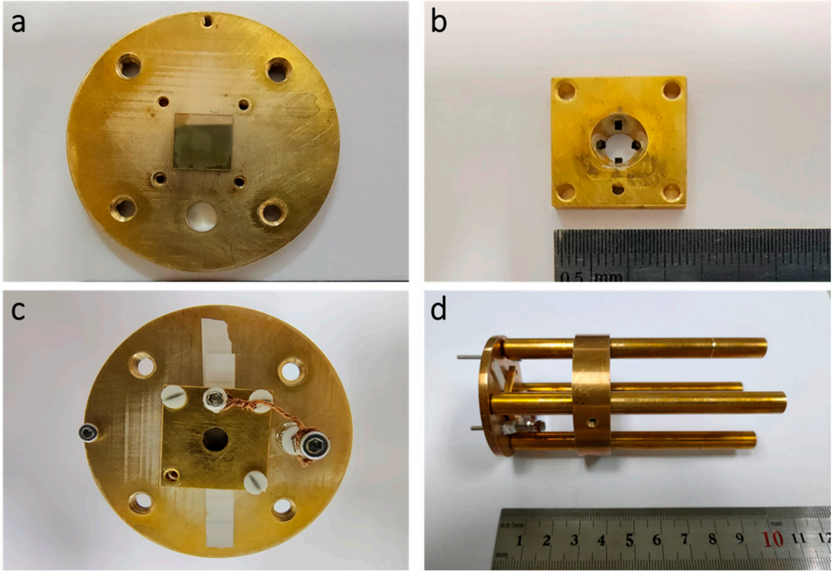

Figure S1. Digital photos of (**a**) the anode component, (**b**) the cathode component, (**c**) the cathode–anode diode driving structure and (**d**) the device with ultraviolet lens to improve light beam divergence.

Figure S1 presents actual component pictures and the assembly process of our designed and fabricated device. The AlN thin film grown on sapphire substrate is attached to the center of the holder with conductive silver paste to form the anode component (Figure S1a). The cathode is fabricated with CNT cubes transferred and evenly distributed on four symmetrical points (Figure S1b). The cathode–anode diode driving structure is shown in Figure S1c, in which the distance between the cathode and anode is controlled by the white insulation gaskets. An ultraviolet lens is placed on the outgoing light path to improve beam divergence (Figure S1d).

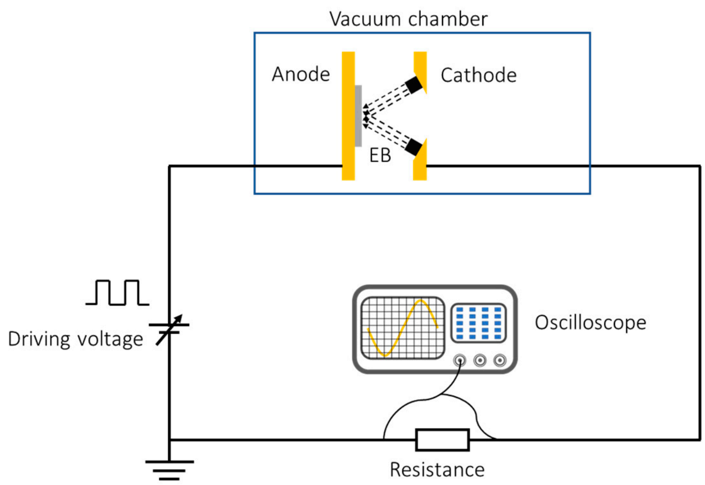

Figure S2. The schematic diagram of the operating circuit.

The operating circuit in this work is shown in Figure S2. The ultraviolet light-emitting device was loaded in a vacuum chamber with a pressure of 1×10^{-6}. Adjustable square high-voltage pulses with a 100 Hz repetition frequency were applied to the anode. The EB emitted from the CNT cold cathode through field emission excited the AlN thin film to generate ultraviolet emission. An oscilloscope with a resistance was used to display the driving voltage waveform and calculate the current.

II. Measurement of Power Efficiency

A schematic diagram of the light output power measurement setup is shown in Figure S3. The emission emitted from the AlN thin film first passed through a 1-inch ultraviolet lens to improve beam divergence, and then exited from the vacuum chamber through a 2-inch sapphire window. A 2-inch ultraviolet lens was placed close to the sapphire window to focus the beam. Finally, the output power was measured by a power meter. When the square driving voltage with a 10% duty ratio was 3.92 kV (namely V_d = 3.92 kV, δ = 10%), the corresponding amplitude of square current was 1.6 mA (I_d = 1.6 mA). Thus, the input electrical power (P_{in}) was about 62 mW, which can be calculated using the expression $P_{in} = I_d V_d \delta^2$. Under this excitation condition, the light output power (P_{out}) was measured to be about 10 μW. Consequently, the power efficiency (PE) of our device was estimated to be about 0.02%.

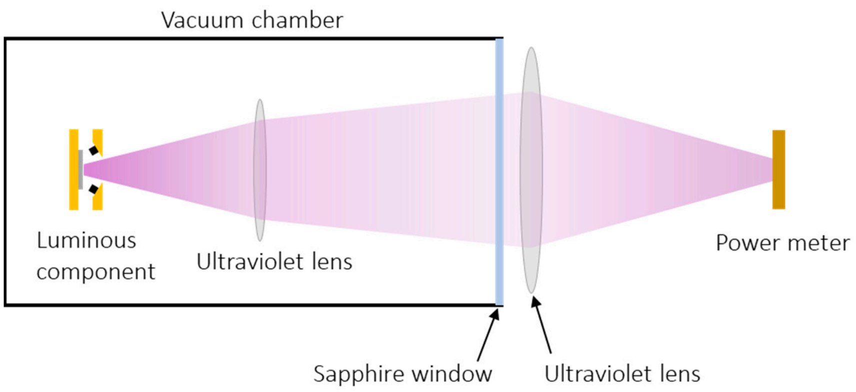

Figure S3. Schematic diagram of the light output power measurement setup.

III. Cathodoluminescence with Different Duty Ratios

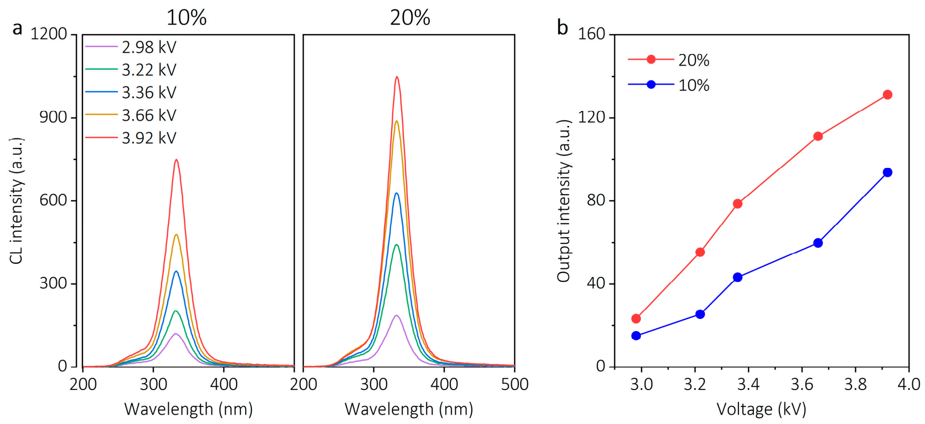

Figure S4. (**a**) CL spectra and (**b**) output intensity of the device for δ = 10%, 20%.

The CL spectra and output intensity of the ultraviolet device for δ = 10%, 20% are shown in Figure S4. For ease of comparison, the CL spectrum under driving voltage of the same amplitude is marked with the same color. It can be observed that the emission is stronger with a higher duty ratio. Under driving voltage of the same amplitude, the electrons emitted from the CNT cold cathode obtain the same energy, but a higher duty ratio allows more electrons to escape from CNTs and participate in the CL process. In an ideal condition, the output intensity with a δ of 20% is twice that with a δ of 10%. However, the electron accumulation caused by the poor conductivity of the AlN thin film may weaken the electron emission. Taking this charge shielding effect and measurement error into consideration, the actual result has a little deviation from the ideal condition. Finally, it can be seen that the output intensity is the result of the combined effect of energy and quantity of electrons.

IV. Simulation of Electron Trajectory

In an EB pumping device, the penetration depth of electrons is an important parameter that deserves attention. In this work, we simulated electron trajectories injected in the AlN sample using software "CASINO_v2.5.1.0", which is based on Monte Carlo method and available at https://www.gegi.usherbrooke.ca/casino/index.html (accessed on 15 March 2023). During the simulation, the thickness of the AlN layer was set as 380 nm and the densities of AlN and sapphire were set as 3.23 g/cm^3 and 4.00 g/cm^3, respectively. A series of simulations have been taken under various driving voltages (V_d = 3 kV, 3.5 kV, 4kV). The results are shown in Figure 6. It can be seen that: (a) a larger driving voltage leads to a larger penetration depth. (b) The penetration depth is less than the thickness of the AlN layer even at the largest driving voltage (V_d = 4 kV), which means almost all electrons contribute to CL emission, instead of taking an energy loss in the substrate.
